# Predictors of weight and waist gain in US South Asians: Findings from the Mediators of Atherosclerosis in South Asians Living in America (MASALA) Study

**DOI:** 10.21203/rs.3.rs-4054151/v1

**Published:** 2024-03-28

**Authors:** Sujatha Seetharaman, Isabel Allen, Meghana Gadgil, Shylaja Srinivasan, Lisa Topor, Alka Kanaya

**Affiliations:** University of California San Francisco; University of California, San Francisco; University of California, San Francisco School of Medicine; Warren Alpert Medical School at Brown University; University of California, San Francisco

**Keywords:** Weight gain, Weight stable, Waist gain, South Asians living in the US, Adiponectin and Leptin

## Abstract

**Background.:**

Weight and waist gain are significant concerns in adulthood. Both weight and waist gain are particularly important among South Asians, a high-risk group known to develop chronic cardiometabolic complications at any body mass index compared to other racial and ethnic groups.

**Objective.:**

The aim of this study was to investigate factors predicting weight and waist gain in a longitudinal cohort of US South Asians, a high-risk group for developing obesity-related complications.

**Methods.:**

We used data from Mediators of Atherosclerosis in South Asians Living in America study (MASALA) exam 1 (2010–2013) and exam 2 (2015–2018), with a mean 4.8 years of follow-up.

**Results.:**

Of 634 participants studied (42.7% women, mean age 55 years, BMI 25.7 kg/m2, weight 70.4 kg at exam 1), 34.7% had gained ≥5% weight and 32.3% gained ≥5% waist at exam 2. In the adjusted models, older age, higher number of years of US residence, and having diabetes were associated with lower odds of weight gain; being female and having higher adiponectin were associated with higher odds of weight gain. Being female, employed full or part time, or retired were associated with lower odds of waist gain. Being single, separated/divorced, having a higher leptin and a higher C-reactive protein level were associated with higher odds of waist gain.

**Conclusions.:**

South Asian subgroups with higher risk of weight and/or waist gain may benefit from targeted interventions to improve health outcomes.

## Introduction

Weight gain is a significant health concern during adulthood. Research has shown that 98% of men and 92% of women experience an upward trajectory with an estimated weight gain of 0.53 kg (1.17 lbs.) per year (1). Adults who gain the most weight have the highest risk for cardiovascular and other obesity-related conditions (1). Not only weight, but the risk for central adiposity as measured by waist circumference (WC), also increases during adulthood, and increases the risk of obesity-related morbidity and mortality (2, 3). Both weight and waist gain are particularly important among South Asians, who have been identified as a high-risk group for developing chronic cardiometabolic complications at any BMI compared to other racial and ethnic groups (4, 5). Understanding factors that predict weight and waist gain are important for prevention efforts in this high-risk population.

Past studies have found that determinants of weight and waist change during adulthood are multifactorial and can be influenced by sociodemographic factors (6–8), dietary factors (9–13), behaviors such as alcohol use and smoking (12, 13), physical activity levels (7, 11), metabolic conditions such as diabetes (6, 14), and psychological factors (15). Similar studies examining weight and waist change over time are limited among South Asian Americans, an underrepresented group in research studies.

Work from the Mediators of Atherosclerosis in South Asians Living in America (MASALA)Study (16) has demonstrated that South Asians have a high-risk phenotype for developing diabetes and hypertension at a lower BMI (17–20) compared to non-Hispanic White, Black, Hispanic, and Chinese American populations. The body composition in South Asians is sometimes termed the “thin fat” phenotype, describing a lower BMI and low lean body mass, with higher amount of visceral fat and adipose tissue in ectopic sites such as the liver and skeletal muscle (21). Therefore, measurement of waist circumference more than weight is very important in this population. In addition to a less favorable body composition, a cross sectional study using the MASALA and Multi Ethnic Study of Atherosclerosis (MESA) data showed that compared to other racial and ethnic groups, US South Asians had lower adiponectin and higher resistin levels, both of which have been implicated in increasing risk for insulin resistance and obesity (18). Less is known about whether body composition, adipokine levels, and metabolic factors influence weight and waist gain in US South Asians.

Addressing paucity of literature, we aimed to prospectively investigate factors associated with weight and waist gain change over time, in a community-based cohort of South Asian Americans, to help elucidate potential subgroups at higher risk for targeted prevention strategies.

### Research Design and Methods

#### Study design

We used data from the baseline exam 1 (2010–2013) and exam 2 (2015–2018) of the MASALA study. Briefly, MASALA is a prospective community-based cohort study of South Asian Americans who were recruited from two clinical sites: the San Francisco Bay Area at the University of California, San Francisco (UCSF), and the greater Chicago area at Northwestern University (NU). Details of the study design, recruitment, and sampling methods have been previously published (16). A total of 906 subjects were recruited between October 2010 and March 2013 and 749 (83%) participants returned to complete exam 2 with a mean 4.8 years of follow-up (16).

#### Ethics

The institutional review boards at both the University of California, San Francisco and Northwestern University approved the study protocol and informed consent was obtained from all participants at each exam. The current analysis was approved by the Institutional review board at the University of California, San Francisco.

#### Study Participants

Eligibility criteria included participants self-identifying with a South Asian background (three out of four grandparents born in any South Asian country), ages between 40–84 years, ability to speak and read English, Hindi, or Urdu, and have no known cardiovascular disease (16). Exclusion criteria included individuals who reported nitroglycerin medication use; had active cancer; shortened life expectancy < 5 years; impaired cognitive ability; plans to move out of the geographic vicinity of the study site in the next 5 years; living in a nursing home; or weighed > 300 lbs. due to computed tomography scanner weight limits (16). Similar procedures for the physical examination and laboratory measures were conducted at baseline and follow-up (16).

Our analytic sample consisted of participants who had complete data on weight, height, and waist circumferences at both exams.

### Study measurements

#### Outcome variables

##### Anthropometry.

Participant’s weight (kg) was measured using a digital weighing scale, and height (cm) was recorded using a stadiometer. BMI (kg/m^2^) was calculated based on participants’ height and weight (16). A continuous BMI measure and a categorical BMI measure were created based on Asian obesity categories by the World Health Organization: Overweight (BMI 23 to < 27.5 kg/m^2^) and obesity (BMI 27.5 kg/m^2^ or more) (22). Waist circumference (WC, cm) was measured using a flexible measuring tape at the level of the umbilicus between the lower ribs and the anterior superior iliac spine (16). A categorical WC measure was created to classify individuals who had abdominal obesity based on Asian category (men ≥ 90 cm and women ≥ 80 cm) (23). Weight and waist circumference change between exams 2 and 1 were calculated.

Participants were categorized as having weight or waist gain (≥ 5% weight or waist gain from exam 1) or being weight or waist stable (± 4.9% weight or waist change from exam 1). Among 748 participants, we excluded 34 (4.5%) with weight loss ≥ 5% and 51 (6.8%) with waist loss ≥ 5% and 29 (3.8%) with both weight and waist loss weight loss ≥ 5% from exam 1, for this analysis, leaving a final analytic sample of 634 participants.

##### Predictor variables

We included self-reported social-demographic information, behavioral and psychological characteristics, and clinical measures that were collected from the participants during their baseline exam by bilingual trained staff (16).

##### Social-demographic variables.

Participant’s age in years, gender (male or female), place of birth (US or outside of US), number of years of US residence for non-US born participants, marital status (single, married, separated or divorced, or widowed), occupational status (not working, working part or full-time, unemployed, or retired), education level (less than, equal to, or greater than a Bachelor’s degree), and health insurance status (yes or no).

##### Behavioral variables.

Tobacco use was assessed by asking participants their smoking status (never, former, or current smoker). Alcohol use was assessed by asking about alcohol drink consumption per week. Sedentary behavior was assessed by asking participants the number of minutes per week they watch television. Physical activity was assessed by asking participants the number of minutes of moderate exercise they did during a week using the Typical Weeks Activity Survey (24). Dietary intake over the previous year was assessed using the Study of Health Assessment and Risk in Ethnic Groups (SHARE) food frequency questionnaire, which has been validated among South Asians in Canada (25). Fasting behaviors were assessed by asking the participants if they fasted once or more per week, once or more per month or once a year or never. Frequency of eating out was assessed by asking if they ate out 2–3 times per week or once or less than once per week.

##### Psychological factors.

Several psychological factors were analyzed that were assessed using Spielberger trait anxiety scale, the Center for Epidemiologic Studies Depression Scale (CES-D), Spielberger anger scale, and chronic psychological burden (26–28).

##### Body Composition.

Abdominal computed tomography (CT) scans (Philips Medical Systems, Andover, MA; Toshiba Medical Systems, Tustin, CA; Siemens Medical Solution, Malvern, PA) were used to determine abdominal visceral fat area and abdominal intermuscular fat area (16). Visceral and subcutaneous abdominal fat were measured at the L4–L5 level using the Medical Image Processing, Analysis, and Visualization (MIPAV) software at the University of California, San Diego body composition reading center (29). Visceral fat was defined as those pixels within the appropriate Hounsfield Unit (HU) range and within the contour of the visceral cavity. The four abdominal/back muscle groups from which abdominal intermuscular fat was measured included the psoas, paraspinous, oblique, and rectus muscles. These muscles were highlighted by the readers and then deleted from the calculation of the subcutaneous fat area.

Fatty Liver disease. CT images for liver fat attenuation (higher attenuation implying lower fat in the liver) were interrogated using the MIPAV software at the vertebral level of T12–L1. Fatty liver was defined at hepatic attenuation ≤ 40 HUs.

##### Adipokines/inflammatory markers.

A panel of blood tests were undertaken to assess the adipokine and inflammatory marker profile of participants that included measurement of leptin, adiponectin, resistin, and high sensitivity C-reactive protein (CRP) levels after a 12-hour fast. Adiponectin and resistin levels were measured using the Millipore Luminex adipokine panel A (EMD Millipore, Billerica, MA). The intraassay coefficient of variations (CV) was 2.34–4.12% for adiponectin and 3.25–5.03% for resistin. Serum leptin levels were measured in duplicate (RIA for total Leptin and ELISA for high molecular weight Leptin; Linco, St Charles, MO, USA) and the intra-assay coefficient of variation was 6.0%.

##### Metabolic profile

Glucose metabolism. A series of tests were conducted to measure dysglycemia in participants including a HbA1c. Among those who were not on any diabetes medicines, an oral glucose tolerance test was conducted to measure glucose and insulin levels. This included a fasting glucose test (measured using the hexokinase method) to determine glucose levels at baseline, followed by a glucose test at 2 h after ingestion of a 75-g glucose challenge. Participant’s glycemic status was defined according to American Diabetes Association criteria. Normal glycemia was defined as having a FPG < 100 mg/dL and 2-hour glucose < 140 mg/dL. Prediabetes was defined as having a fasting plasma glucose between 100 to 125 mg/dL or 2-hour glucose of 140 to 199 mg/dL. Type 2 diabetes was defined as use of diabetes medications, or fasting plasma glucose ≥ 126 mg/dL, and/or a 2-hour post challenge glucose ≥ 200 mg/dL.

Fasting serum samples were batched for insulin, measured by the sandwich immunoassay method (Roche Elecsys 2010; Roche Diagnostics, Indianapolis, IN). The homeostasis model assessment (HOMA)-IR was used to measure IR and calculated as [Insulin_0_(mIU/mL) x Glucose_0_ (mmol/L)/22.5]. The homeostasis model assessment of Beta cell function (HOMA-b) which was used calculated as [20 x Insulin0(mIU/mL)/Glucose0(mmol/L)-3.5].

Lipid panel. A fasting lipid profile was obtained that included total cholesterol, triglycerides, calculated low-density lipoprotein (LDL), and high-density lipoprotein (HDL) levels.

Blood pressure. Seated resting blood pressure was measured three times using an automated blood pressure monitor (V100 Vital Signs Monitor; GE Healthcare, Fairfield, CT) and the average of the last two readings were used for analysis (16). Hypertension was defined as a systolic blood pressure of 140 mm Hg or greater and/or diastolic blood pressure of 90 mm Hg or greater or use of antihypertensive medication.

### Statistical analysis

Initially, we generated univariate summaries with frequencies and percentages for categorical variables and means, median, standard deviations, minimum, and maximum for continuous variables. Bivariate relationships were examined between all potential exposure variables with participants’ weight and waist circumference change. Chi-squared statistics were calculated between categorical variables, Analysis of Variance and Student’s t-tests were used to compare continuous variables, and correlations were calculated between continuous variables. Stepwise multivariable logistic regression methods were used to examine the association of weight gain using metabolic, body composition, psychological factors, behaviors, and sociodemographic factors from exam 1, after adjusting for age, gender, baseline BMI, and years lived in the US. Similarly, we performed stepwise linear regression methods using to examine the prospective association of waist circumference change in exam 2 using exposures from exam 1, after adjusting for age, gender, marital and occupational status. We also explored pertinent interaction effects in the adjusted models for whether gender, baseline BMI status, and glucose tolerance status modified the effect of any of the significant risk factors for weight and waist gain. Analysis was performed using Stata software, version 17 (Stata Corp. 2021, College Station, TX).

## Results

Among the 634 participants studied (42.7% women, mean age 55 years, BMI 25.7 kg/m^2^, weight 70.4 kg at exam 1), 414 (65.3%) were weight stable (± 4.9% weight change) and 220 (34.7%) had gained ≥ 5% weight from exam 1; 429 (67.7%) participants were waist stable (± 4.9% waist change) and 205 (32.3%) participants had gained ≥ 5% waist from exam 1 (Table 1).

Bivariate analysis associated with weight increase included having a higher adiponectin per SD (β 0.31; 95% 0.06–0.56; p = 0.016). Factors associated with weight decrease included retired work status (β 1.22; 95% CI −1.94- −0.49; p = 0.001), having one alcoholic drink per week (β 0.52; 95% CI −1.04- −0.01; p = 0.046), having prediabetes (β 0.42; 95% CI −1.03– 0.20; p = 0.183), having diabetes (β 2.09; 95% CI −2.81- −1.37; p < 0.001), or having hypertension (β 0.83; 95% CI −1.33 −0.33; p = 0.001) (Table 1).

Bivariate analyses associated with waist gain included being separated or divorced (β 2.95; 95% CI 0.10–5.8; p = 0.043) or being widowed (β 7.60; 95% CI 3.47–11.73; p < 0.001); being unemployed (β 3.03; 95% CI 1.53–4.53; p < 0.001), having a higher emotional burden (β 0.45; 95% CI 0.004–0.91; p = 0.048) or having a higher CRP level (β 0.30; 95% CI 0.18–0.41; p < 0.001). Waist decrease was associated with being a former smoker (β 1.72; 95% CI −3.06- −0.38; p = 0.012) or having one alcoholic drink per week (β 1.48; 96% CI −2.45- −0.51; p = 0.003).

### Supplementary Description

Supplementary Table 1 shows adipokine and CRP levels by gender, glycemic status, and Asian BMI category. We found that adiponectin, leptin and CRP levels were higher in females compared to males. However, we found that while adiponectin levels were higher among those with a normal BMI and without diabetes, leptin and CRP levels were higher in individuals with overweight, obesity, with prediabetes and diabetes (supplemental Table 1).

### Predictors of weight gain

In the adjusted logistic regression models, higher number of years of US residence (OR 0.98; 95% CI 0.96–0.99, p = 0.041) and having diabetes (OR 0.38; 95% CI 0.21–0.70; p = 0.002) at exam 1 were associated with lower odds of weight gain in exam 2. Female sex (OR:1.51; 95% CI 1.02–2.24; p = 0.040) and higher adiponectin (OR 1.39 per SD; 95% CI 1.13–1.71; p = 0.002) were associated with higher odds of weight gain (Table 2).

Linear regression analysis also showed that having diabetes was associated with clinically meaningful weight loss in exam 2 (ß −1.60; 95% CI −2.39- −0.82; p < 0.001) (Table 2). Older age (ß 0.05; 95% CI −0.08- −0.02; p = 0.004) and higher adiponectin level (ß 0.34 per SD; 95% CI 0.05–0.62; p = 0.019) were associated with weight gain in exam 2 (Table 2).

We examined associations between being on diabetes medications (metformin, insulin) in exam 1 and weight change in exam 2. We found that among the 624 individuals, 77 were taking metformin and 10 were taking insulin for diabetes. Among those on metformin, 65 individuals (84.42%) were in the weight stable group in exam 2. Only 2 (20%) individuals on insulin were in the weight gain group in exam 2.

### Predictors of waist gain

Logistic regression models showed that being employed full/part time (OR 0.57; 95% CI 0.35–0.94; p = 0.027) or being retired (OR 0.51; 95% CI 0.26–1.02; p = 0.056) were associated with a lower odds of waist increase (Table 3), and only female gender (OR1.82; 95% CI 1.16–2.87; p = 0.009) was associated with greater odds of waist increase (Table 3).

In the adjusted linear regression models, being employed full/part time (ß −1.32; 95% CI −2.70– 0.05; 0.059) was associated with a decrease in waist (Table 3). Being separated/divorced (ß 2.69 95% CI −0.08–5.47; p = 0.057), being single (ß 4.90; 95% CI 0.79–9.01; p = 0.019), having a higher leptin level (ß 0.72; 95% CI 0.06–1.38; p = 0.032), and a higher CRP level (ß 0.82; 95% CI 0.34–1.29; p = 0.001) were associated with waist increase (Table 3).

### Interaction testing

We found important evidence for interactions for weight gain. Men with obesity at baseline had a lower likelihood of weight gain compared to women with normal BMI. Regardless of glucose tolerance status, individuals with overweight and obesity had a lower likelihood of weight gain compared to those with a normal BMI (Table 2). Individuals with overweight and obesity, who were in the waist stable group had lower weight gain compared to normal weight individuals in the waist gain group (Table 2).

For waist gain interactions, we found that regardless of gender, individuals with overweight and obesity had a lower waist gain compared to those with a normal BMI (Table 3). Females with a higher CRP level and higher leptin level had a greater waist increase in exam 2. Regardless of baseline BMI, all individuals with weight gain also had waist gain.

## Discussion

This is the first study to prospectively examine factors influencing weight and waist change in a cohort of US South Asians. After adjusting for demographic characteristics, female gender and higher adiponectin levels were associated with a greater likelihood of weight gain, while older age, higher number of years of US residence, and having diabetes were associated with a lower odds of weight gain. In contrast, having higher leptin and CRP level and being separated/divorced or single were associated with a greater waist circumference change, while being employed full or part time was associated with lower odds of waist gain. Individuals with weight gain also had waist gain, irrespective of their baseline BMI. In contrast, individuals with BMI in the overweight or obesity range at baseline who were in the waist stable group had a decrease in weight in exam 2.

We found a significant effect of adipokines and CRP levels on weight change. Higher baseline adiponectin levels were associated with weight gain. Adiponectin, a “beneficial and anti-inflammatory adipokine”, is thought to be protective against insulin resistance and type 2 diabetes (30). Prior studies to characterize relationship between adiponectin levels and subsequent weight change have not seen significant associations except for the Nurses’ Health Study that demonstrated that higher adiponectin levels were positively associated with weight gain in relatively “healthy” women who did not subsequently develop diabetes during a long follow-up period. Though other studies did not find this same association between adiponectin and weight gain, these studies were conducted in specific populations such as Pima Indians (31), Afro Jamaican adults (32), and elderly White populations (33) and our current study on South Asians Americans. Further, the Nurse’s Health Study was limited by use of BMI calculated from self-reported weight and height and included mostly “healthy” women without prior diagnosis of chronic disease such as diabetes (34). Adipokine levels varied by gender, BMI, and glycemic status in the current study. We found that females without diabetes and with a normal BMI had the highest baseline adiponectin levels. Similar to the results of the Nurse’s Health study, we found that the relatively “healthy” females without diabetes and normal BMI were most likely to gain weight. These findings on the association between adiponectin and weight gain are hypothesis generating and future studies are required to determine the utility of adiponectin as a potential biomarker for weight change in any population. Additionally, associations of higher leptin and CRP levels with waist gain have been identified in prior studies (35–38) in multiple racial and ethnic groups including South Asians and are consistent with our findings. Thus, higher leptin and CRP levels might be potential biomarkers in the future for waist gain, representing future cardiovascular and metabolic risk.

In the current study, findings were not consistent across all weight groups or by gender. We found that women were at risk for both weight and waist gain compared to men. Additionally, we found that men with both overweight and obesity had a decreased likelihood of weight gain compared to women in any of the BMI categories, placing women at an increased risk for developing obesity-related comorbidities such as type 2 diabetes. These findings contrast with a prior study by Jackson et al., using a large cohort of individuals in the US Health and Retirement Study and the UK English Longitudinal Study of Ageing that found that women were more likely to have weight loss compared to men (39). These findings indicate that the trajectories of weight and waist change may be different in middle- and older-aged South Asian men and women and further studies are required to explore these differences. These findings may have potential implications for gender-specific strategies for management of weight, particularly in middle and older aged South Asian women.

Our study findings of weight decrease among individuals with diabetes was similar to past studies that have showed that a diagnosis of type 2 diabetes in adults is associated with progressive weight loss following the diabetes diagnosis (40, 41). Medications used for diabetes (such as metformin that can cause modest weight loss in some individuals) could explain the improved trajectory in some these individuals since majority of the individuals on metformin were in the weight stable group. There was no significant effect of insulin use on weight change in this study. In the current study, we found that independent of glucose tolerance status, individuals with obesity were less likely to gain weight and so were the overweight individuals with prediabetes. A past study by Jackson et al (39) reported that individuals with obesity had a higher likelihood of clinically meaningful weight loss in the future. It is possible that individuals with obesity or overweight and with diabetes had more frequent health checks, were more mindful of their weight status and thus were adopting healthy lifestyle behaviors and/or seeking pharmacological treatments to manage their weight and prevent future weight gain.

These findings have implications for obesity management intervention efforts for South Asians, a high-risk group for developing obesity-related complications. The observed effects of social capital such as marital status and occupational status on waist change are important as these social factors may facilitate or hinder adoption of healthy lifestyle behaviors. These findings are relevant for health promotion efforts as targeted interventions for these higher-risk individuals may reduce development of central adiposity and, by extension, the occurrence of obesity and its health consequences.

Study findings should be considered in light of several limitations. Firstly, the MASALA study is representative of primarily middle-aged to older Asian Indian immigrants in the US, and thus is not generalizable to other South Asian subgroups in the US. Secondly, the MASALA study excluded individuals with a body weight greater than 300 lbs. due to CT scanner capacity and thus limiting our ability to assess weight and waist gain patterns in these individuals with the highest levels of obesity. Thirdly, the population in this study was homogenous with high socioeconomic attainment, limiting generalizability to individuals with lower educational or income. The duration of being overweight or having obesity and data regarding intentional versus unintentional weight loss or gain and participation in a weight control program were not collected in the study. Offsetting these limitations were several strengths. The MASALA study is the first and the largest study in South Asians in the US to prospectively evaluate weight and waist gain in South Asians. The study utilized systematic and standardized data collection including use of measured weight and height to calculate accurate BMI. In addition to clinical, behavioral, and psychological measures, the MASALA study included other important variables such as several radiographic measures of body composition, adipokine levels, and metabolic conditions such as diabetes, fatty liver disease, and hypertension.

## Conclusions

In conclusion, in a prospective cohort of US South Asians, we found that higher adiponectin levels and female gender were associated with a greater odds of weight gain, whereas older age, greater years of US residence, and having diabetes were associated with lower odds of weight gain. Female gender, being separated, divorced, or single, having higher leptin and CRP levels were associated with waist gain, while being employed full or part time was associated with a lower odds of waist gain. In addition to identifying several social, demographic, and clinical factors that can serve as targets for obesity interventions in this high-risk population, the current study also raises hypotheses about associations of adipokine levels with weight and waist gain.

## Figures and Tables

**Figure 1:**
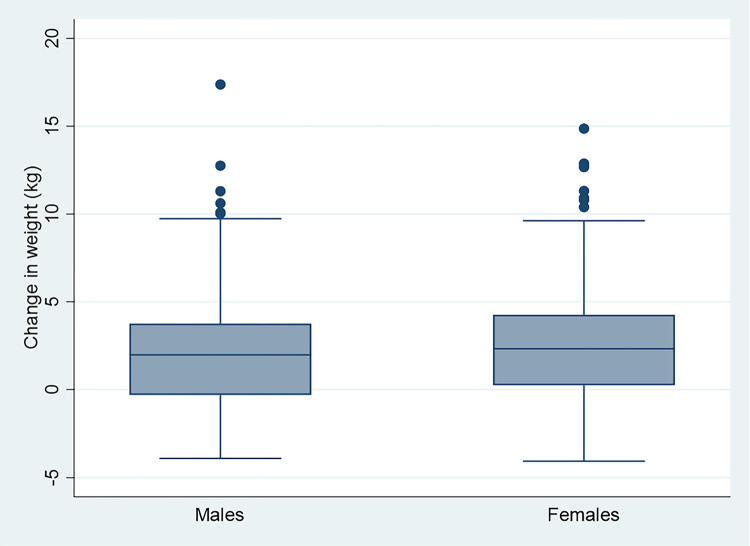
Graph showing change in weight by sex between exam 1 and exam 2

**Figure 2:**
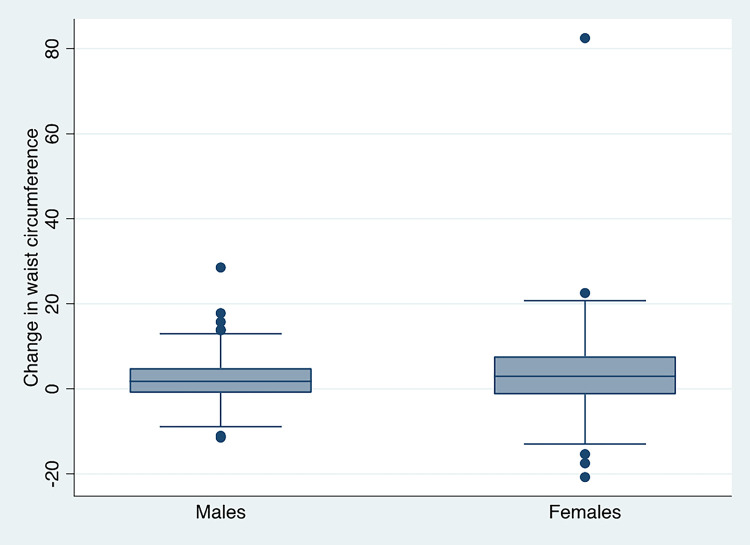
Graph showing change in waist by sex between exam 1 and exam 2

**Table 1:** MASALA participant characteristics by weight and waist change groups, 2010–2013 (N = 634)

Anthropometry	Weight Stable ± 4.9% weight change* n = 414 (65.3%)	Weight gain > 5% weight gain* n = 220 (34.7%)	Waist Stable ± 4.9% waist change* n = 429 (67.7%)	Waist gain > 5% waist gain* n = 205 (32.3%)
**BMI (by Asian Category) at exam 1 Normal BMI (BMI < 23)**	**84 (20.3%)**	**72 (32.7%)**	**103 (24.0%)**	**53 (24.6%)**
Overweight (BMI 23-<27.5)	202 (48.8%)	100 (45.5%)	210 (48.9%)	92 (44.9%)
With obesity (BMI 27.5 or more)	128 (30.9%)	48 (21.8%)	116 (27.0%)	60 (29.3%)
Weight at exam 2 (kg)	71.9 (64.2–79.2)	72.3 (63.3–88.8)		
Waist circumferance (cm) at exam 2	95 (89-. 5–101.9)	94.3 (88.4–103.4)		
BMI (kg/m2) at exam 2	25.9 (23.8–28.6)	26.5 (24.2–29.6)		
Absolute Weight change (kg)	0.7 (1.9)	5.6 (2.3)	1.7 (2.7)	3.9 (3.5)
Absolute Waist circumferance change (cm)	2.2 (6.0)	5.9 (5.2)	0.6 (2.6)	9.6 (6.5)
Absolute BMI change (kg/m^2^)	0.4 (1.1)	2.2 (1.1)	0.7 (1.2)	1.6 (1.6)
**Predictor variables**				
**Sociodemographic characteristics Age at exam 2** (years)	60.4 (9.3)	57.5 (9.2)	59.2 (9.3)	59.7 (9.5)
**Female**	150 (36.2%)	121 (55%)	157 (36.6%)	114 (55.6%)
**Place of birth** India	340 (82.1%)	191 (86.8%)	358 (83.5%)	173 (84.4%)
Other South Asian country	64 (15.5%)	22 (10.0%)	58 (13.5%)	28 (13.7%)
Other diaspora country	10 (2.4%)	7 (3.2%)	13 (3.9%)	4 (1.9%)
**Years lived in the US**	28.3 (11.0)	25.1 (10.8)	27.4 (10.9)	26.8 (11.3)
**Education** <= High school	25 (6.0%)	11 (4.8%)	24 (5.6%)	11 (5.4%)
< bachelor's degree	20 (4.8%)	8 (3.5%)	19 (4.4%)	8 (3.9%)
≥bachelor's degree	369 (89.1%)	406 (91.7%)	386 (89.9%)	186 (90.7%)
**Marital status** Married/living as married/with partner	381 (92.0%)	20 (92.3%)	400 (93.2%)	184 (89.8%)
Separated/divorced	9 (2.2%)	8 (3.7%)	8 (1.9%)	9 (4.4%)
Widowed	17 (4.1%)	8 (3.7%)	15 (3.5%)	10 (4.9%)
Single	7 (1.7%)	1 (0.5%)	6 (1.4%)	2 (0.9%)
**Occupation** Not working/unemployed	56 (13.5%)	39 (17.7%)	48 (11.2%)	47 (22.9%)
Employed full-time/part-time	290 (70.0%)	161 (73.2%)	320 (74.6%)	131 (63.9%)
Retired	68 (16.4%)	20 (9.1%)	61 (14.2%)	27 (13.2%)
**Health Insurance**	384 (92.8%)	209 (95.4%)	401 (93.5%)	192 (94.1%)
**Behavioral characteristics Cigarette smoking status** Never/Former smoker	399 (96.4%)	214 (97.3%)	412 (96.0%)	201 (98%)
Current smoker	15 (3.6%)	6 (2.7%)	17 (3.7%)	4 (1.9%)
**1 + alcoholic drinks/week**	164 (39.6%)	60 (27.3%)	163 (38.0%)	61 (29.8%)
**TV watching, minutes/week**	420 (210–840)	420 (210–840)	420 (210–840)	420 (210–840)
Moderate physical activity (MET-min/week)	2906.3 (1635–4920)	3097.5 (1518.8–5302.5)	2992.5 (1620–5100)	2940 (1530–4702.5)
**Vegetarian diet**	142 (34.8%)	90 (41.2%)	152 (36.0%)	80 (39.4%)
**Fasting** About once or more/week	36 (8.7%)	31 (14.1%)	45 (10.5%)	22 (10.7%)
About 1–2 times/month	69 (16.7%)	27 (12.3%)	66 (15.4%)	30 9 (14.6%)
Once a year/or almost never	309 (74.6%)	162 (73.6%)	318 (74.1%)	153 (74.6%)
**Eating out** 2 or 3 times a week	44 (10.6%)	33 (15%)	50 (11.7%)	27 (13.2%)
Once or < once a week	370 (89.4%)	187 (85.0%)	379 (88.3%)	178 (86.8%)
**Psychological characteristics** Spielberger anger score	15.8 (3.7)	16.1 (3.7)	15.8 (3.8)	16.2 (3.6)
CES-Depression score	7.1 (6.4)	7.8 (7.5)	7.0 (6.6)	8.0 (7.1)
Social support score	25.1 (4.8)	24.7 (4.9)	25.1 (4.7)	24.5 (5.1)
Emotional burden score	0.8 (1.0)	0.9 (1.1)	0.8 (1.0)	0.9 (1.1)
Spielberger anxiety score	15.8 (4.3)	15.9 (4.5)	15.8 (4.4)	16.0 (4.2)
**Body composition measures** Abdominal subcutaneous fat area (cm^2^)	235.9 (95.4)	224.7 (89.4)	229.8 (90.0)	236.7 (100.4)
Abdominal visceral fat area (cm^2^)	141.7 (50.9)	120.7 (57.2)	134.8 (52.4)	133.6 (57.4)
Liver attenuation (HU)	54.1 (10.4)	57.6 (10.2)	54.8 (9.7)	56.3 (11.8)
Intermuscular fat area (cm^2^)	21.8 (8.9)	19.8 (7.9)	20.8 (8.7)	21.9 (8.2)
Abdominal lean muscle mass area (cm^2^)	97.0 (24.5)	90.9 (23.8)	97.0 (24.5)	89.1 (23.7)
**Adipokines/inflammatory markers** Leptin (pg/ml)	15668 (13117)	15056 (11755)	14511 (11374)	17463 (14851)
Adiponectin level (ng/dL)	10848 (6429.4)	13276 (6774.1)	11170 (6774.0)	12798 (6242.2)
Leptin (pg/ml)	15668 (13117)	15056 (11755)	14511 (11374)	17463 (14851)
Resistin level (pg/dL)	21563 (10025.9)	21213 (8056.2)	21513 (9827.6)	21289 (8384.9)
CRP level (ug/mL)	2.3 (4.1)	2.4 (3.8)	2.3 (3.9)	2.6 (4.1)
**Metabolic characteristics** Prediabetes	224 (54.1%)	131 (59.6%)	238 (55.5%)	117 (57.1%)
Diabetes	122 (29.5%)	25 (13.3%)	111 (25.9%)	36 (17.6%)
Hypertension	181 (43.7%)	59 (26.8%)	167 (39.9%)	73 (35.6%)
Fatty Liver	44 (10.7%)	12 (5.5%)	39 (9.2%)	17 (8.3%)
Total cholesterol (mg/dL)	186 (162–208.5)	182.5 (163–209.5)	187 (162–209)	183 (162–209)
LDL (mg/dL)	110 (89–133)	109 (90–134)	111 (90–135)	107 (88–129)
HDL (mg/dL)	47 (39–55)	50.5 (41–59)	47 (40–55)	50 (40–59)
Triglycerides (mg/dL)	125 (93–160)	104.5 (78.5–144)	120 (88–158)	112 (88–150)
Insulin (pmol/L)	61 (45–92.9)	49.9 (34.5–75)	59 (41.8–87.9)	55.6 (85.8)
Homeostatic Model Assessment for Insulin Resistance (HOMA-IR) (pmol/L* mg/dL)	2.6 (1.8–4.0)	1.9 (1.3–3.1)	2.4 (1.7–3.7)	2.3 (1.5–3.7)
Homeostasis model assessment of Beta-cell function (HOMA-B) ([pmol/L]/ [mg/dL])	108.4% (69.6–158.9)	99.8% (74.6–140.4)	102.9 (70.2–148.1)	107.4 (71.6–153.8)

All values represent mean ± SD or n (%), or median (IQR) as appropriate.

* Weight and waist change from exam 2 to exam 1

**Table 2:** Multivariable Stepwise Binary Logistic and Linear Model for predictors of Weight Change Among South Asian Americans in the MASALA Study

Variable	Logistic regression for Weight Change ≥ 5%		Linear regression for weight increase (per kg)	
	aOR (95% CI)	p-value	Beta coefficient (95% CI)	p-value
**Age**	0.98 (0.96–1.01)	0.100	**0.05 (−0.08–−0.02)**	**0.004**
**Female Sex**	**1.51 (1.02–2.24)**	**0.040**	0.13 (−0.42–0.67)	0.650
**BMI at exam 1**	0.96 (0.91–1.01)	0.157	0.02 (−0.04–0.09)	0.489
**Years lived in the US**	**0.98 (0.96–0.99)**	**0.041**	-0.01 (−0.04–0.01)	0.381
**Glucose tolerance status:** normal Prediabetes	Reference 0.85 (0.54–1.33)	0.475	Reference −0.23 (−0.870.41)	0.483
Diabetes	**0.38 (0.21–0.70)**	**0.002**	**−1.60 (−2.39–−0.82)**	**<0.001**
**Hypertension**	0.70 (0.45–1.06)	0.087	−0.19 (−0.75–0.37)	0.513
**Adiponectin (per SD)**	**1.39 (1.13–1.71)**	**0.002**	**0.34 (0.05–0.62)**	**0.019**
**Interaction testing**				
BMI** **category x sex** Overweight males	**0.42 (0.22–0.82)**	**0.011**	−0.73 (−1.61–0.15)	0.104
Overweight females	0.51 (0.24–1.07)	0.076	−0.60 (−1.64–0.45)	0.262
Males with obesity	**0.19 (0.06–0.57)**	**0.003**	**–1.87 (−3.26–−0.47)**	**0.009**
Females with obesity	0.38 (0.13–1.16)	0.089	−1.08 (−2.58–0.43)	0.161
**Normal BMI**	Reference		Reference	
**BMI** **x glucose tolerance Overweight, no diabetes	0.56 (0.22–1.41)	0.216	0.07 (−1.22–1.37)	0.911
Overweight, prediabetes	**0.36 (0.18–0.70)**	**0.003**	**−1.04 (−1.95–0.14)**	**0.024**
Overweight, diabetes	0.66 (0.21–2.05)	0.475	−0.54 (−1.92–0.84)	0.443
Obesity, no diabetes	**0.17 (0.04–0.65)**	**0.010**	**−2.39 (−4.18–−0.59)**	**0.009**
Obesity, prediabetes	0.35 (0.12–1.01)	0.052	−1.05 (−2.44–0.35)	0.141
Obesity, diabetes	**0.10 (0.02–0.54)**	**0.007**	**−2.18 (−3.99–−0.38)**	**0.018**
Normal BMI and no diabetes	Reference	Reference		
BMI** **x adiponectin (per SD)** Overweight	**0.74 (0.58–0.93)**	**0.010**	−0.21 (−0.53–0.10)	0.176
Obesity	**0.65 (0.43–0.97)**	**0.034**	−0.35 (−0.89–0.18)	0.199
Normal BMI	Reference	Reference	Reference	Reference
**Adiponectin x sex**				
Females	0.80 (0.54–1.18)	0.262	0.07 (−0.46–0.60)	0.794
Males	Reference	Reference		
**BMI** **x waist change groups (stable versus gain)				
Normal BMI and waist gain	**2.99 (1.41–6.39)**	**0.004**	**1.31 (0.35–2.27)**	**0.007**
Overweight and waist stable	**0.44 (0.23–0.84)**	**0.013**	**−0.91 (−1.71–−0.11)**	**0.025**
Overweight and waist gain	1.57 (0.76–3.25)	0.224	**4.59 (0.48–2.33)**	**0.003**
Obesity and waist stable	**0.20 (0.07–0.61)**	**0.005**	**–1.06 (−3.10–−0.54)**	**0.005**
Obesity and waist gain	1.06 (0.34–3.27)	0.918	4.94 (−0.89–1.92)	0.469
Normal BMI and waist stable	Reference	Reference		

aOR: Adjusted odds ratios from multivariable logistic regression models adjusting for age, sex, BMI, number of years lived in the US at exam 1

BMI**: by Asian BMI category

**Table 3:** Multivariable Stepwise Linear and Binary Logistic Model for predictors of Waist Change Among South Asian Americans in the MASALA Study

	Logistic regression for Waist gain ≥5%		Linear regression for increase in waist (cm)	
Variable	aOR (95% CI)	p-value	Beta Coefficient (95% CI)	p-value
**Age**	1.02 (0.99–1.04)	0.171	0.002 (−0.06–0.06)	0.932
**Female sex**	**1.82 (1.16–2.87)**	**0.009**	1.07 (−0.14–2.27)	0.083
**BMI**	1.00 (0.94–1.06)	0.966	−0.04 (−0.19– −0.12)	0.632
**Marital status**				
Separated/divorced	2.26 (0.84–6.07)	0.106	**2.69 (−0.08–5.47)**	**0.057**
Widowed	0.95 (0.38–2.35)	0.911	0.40 (−2.10 –2.90)	0.753
Single	0.52 (0.09–2.80)	0.449	**4.90 (0.79–9.01)**	**0.019**
**Occupational status**				
Employed full-time/part-time	**0.57 (0.35–0.94)**	**0.027**	**−1.32 (−2.70– 0.05)**	**0.059**
Retired	**0.51 (0.26–1.02)**	**0.056**	−1.07 (−2.91–0.75)	0.255
**Leptin (per SD)**	1.06 (0.83–1.34)	0.664	**0.72 (0.06–1.38)**	**0.032**
**CRP (per SD)**	1.01 (0.85–1.20)	0.929	**0.82 (0.34–1.29)**	**0.001**
**Interaction testing**				
BMI** **x sex**				
Overweight males	0.87 (0.44–1.71)	0.680	**−1.55 (−3.22–0.13)**	**0.070**
Overweight females	0.77 (0.38–1.56)	0.461	**−2.14 (−4.11 - −0.18)**	**0.032**
Males with obesity	1.27 (0.46–3.52)	0.652	**−3.35 (−5.98 - −0.73)**	**0.012**
Females with obesity	0.64 (0.23–1.84)	0.411	**−3.45 (−6.28 - −0.62)**	**0.017**
**BMI** ** **x leptin (per SD)**				
Overweight individuals	0.77 (0.45−1.31)	0.337	−0.95 (−2.39–0.50)	0.198
Individuals with obesity	0.78 (0.42−1.44)	0.430	−0.56 (−2.23–1.11)	0.508
**Leptin x sex**				
Females	1.10 (0.66–1.82)	0.719	**1.62 (0.36–2.89)**	**0.012**
Males	Reference		Reference	
BMI** **x CRP**				
Overweight individuals	1.59 (0.68–3.71)	0.287	0.62 (−0.46–1.70)	0.262
Individuals with obesity	1.51 (0.61–3.74)	0.377	**1.65 (0.55–2.76)**	**0.003**
**CRP x sex**				
Females	0.94 (0.67–1.33)	0.740	**1.27 (0.34–2.19)**	**0.007**
Males	Reference		Reference	
**BMI** **x weight change (gain vs. stable)				
Normal BMI and weight gain	**3.29 (1.60–6.78)**	**0.001**	**3.45 (1.70–5.19)**	**<0.001**
Overweight and weight stable	0.96 (0.46–1.99)	0.907	−1.30 (−2.93–0.33)	0.118
Overweight and weight gain	**3.49 (1.63–7.48)**	**0.001**	**2.34 (0.52–4.16)**	**0.012**
Obesity and weight stable	1.06 (0.37–3.07)	0.911	**−2.62 (−5.08 - −0.15)**	**0.037**
Obesity and weight gain	**6.23 (1.90–20.41)**	**0.003**	1.31 (−1.55– 4.16)	0.369
Normal BMI and weight stable	Reference		Reference	

aOR: Adjusted odds ratios from multivariable logistic regression models adjusting for age, sex, BMI, marital and occupational status in exam 1.

BMI**: by Asian BMI category

## Abbreviations

WC: Waist circumference
BMI: Body mass index
MASALA: Mediators of Atherosclerosis in South Asians Living in America
